# ^18^F-FDG PET/CT Imaging of Idiopathic Granulomatous Mastitis

**DOI:** 10.1097/RLU.0000000000004980

**Published:** 2023-11-25

**Authors:** Keerthini Muthuswamy, Manil Subesinghe

**Affiliations:** From the ∗ King’s College London & Guy’s and St. Thomas’ PET Centre; † Department of Cancer Imaging, School of Biomedical Engineering and Imaging Sciences, King’s College London, London, United Kingdom.

**Keywords:** ^18^F-FDG, PET/CT, granulomatous mastitis, inflammation

## Abstract

A 43-year-old woman, who presented with a suspected left breast abscess, underwent serial ultrasounds, which demonstrated inflammatory changes that were nonresponsive to antibiotics and which spread to the contralateral breast. ^18^F-FDG PET/CT demonstrated diffuse heterogeneous intense FDG uptake in both breasts with reactive axillary nodes. Breast biopsy confirmed granulomatous inflammation, and overall findings were consistent with idiopathic granulomatous mastitis. In the absence of histological analysis, idiopathic granulomatous mastitis is an important differential diagnosis to consider for bilateral abnormal breast uptake, and early recognition can facilitate prompt commencement of treatment.

**FIGURE 1:**
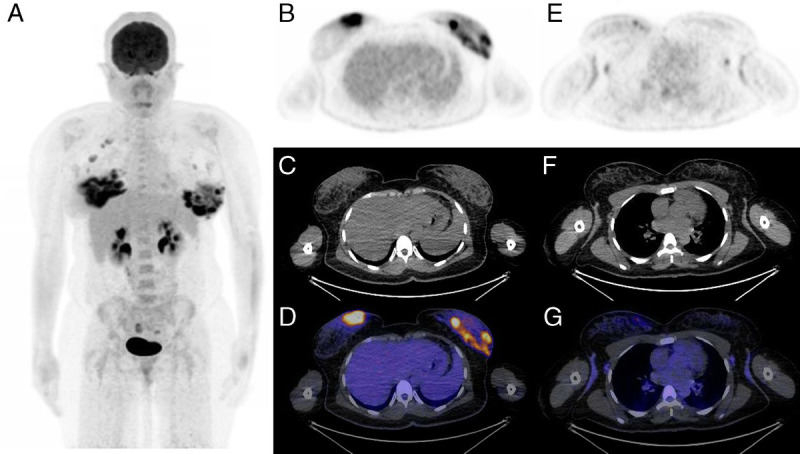
A 43-year-old woman presented with a suspected left breast abscess. Serial ultrasounds demonstrated inflammatory change with areas of mixed echogenicity, dilated ducts, and inspissated material, initially confined to the left breast, which did not respond to antibiotic treatment. These inflammatory changes evolved and extended to involve the contralateral breast along with the development of small loculated collections. Core biopsy confirmed granulomatous inflammation in both breasts with no evidence of malignancy. An ^18^F-FDG PET/CT was performed to assess for a generalized systemic inflammatory disorder, for example, sarcoidosis, that could account for these findings. MIP PET image (**A**), axial PET image (**B**), unenhanced axial CT image (**C**), and axial fused PET/CT image (**D**) demonstrate diffuse heterogeneous intense FDG uptake in both breasts (right > left) associated with areas of increased soft tissue attenuation on CT. Axial PET image (**E**), unenhanced axial CT image (**F**), and axial fused PET/CT image (**G**) through the upper thorax demonstrate bilateral moderately FDG-avid reactive-appearing axillary lymph nodes (right > left). Apart from a reactive-appearing marrow, the remainder of FDG biodistribution is physiological. In the absence of further FDG-avid nodes, specifically within the chest, and no pulmonary parenchymal abnormality, a diagnosis of idiopathic granulomatous mastitis (IGM) was confirmed. IGM is a rare benign chronic inflammatory, usually affecting women of child-bearing age or those with a history of oral contraceptive use.^1–3^ It is typically unilateral; bilateral involvement is reported in 1% to 11% of cases.^4,5^ Clinical and imaging findings across all modalities can be nonspecific and mimic those of malignancy and infection/inflammation due to another cause, thus necessitating prompt core biopsy to establish the diagnosis and commence treatment.^2,3,6–9^ Definitive diagnosis of IGM requires the presence of characteristic histopathologic features (noncaseating lobulocentric granulomatous inflammation) in the absence of other granulomatous conditions such as sarcoidosis, tuberculosis, plasma cell mastitis, and granulomatosis with polyangitis.^2^ Although rare, imaging appearances of IGM on conventional breast imaging (ultrasound and mammography) and breast MRI are well reported.^2–6,8,10–15^ However, ^18^F-FDG PET/CT findings in granulomatous mastitis are sparsely reported with only 1 prior case report demonstrating unilateral IGM mimicking malignancy.^16^ Although ^18^F-FDG PET/CT is not indicated routinely in the diagnosis of IGM, an awareness of this pathology in the correct clinical context together with histopathological findings can facilitate prompt commencement of treatment, which comprises glucocorticoid therapy and regular imaging follow-up^2,4,17^; this is distinct to the management of other pathologies that IGM may mimic. Furthermore, an absence of features suggestive of generalized systemic granulomatous inflammation can also be a diagnostic clue for IGM rather than rarer entities such as isolated intramammary sarcoidosis,^2,18^ sclerosing adenosis,^19^ or progressive transformation of germinal centers within intramammary nodes.^20^
